# A High‐Throughput Hydroponic Assay for Rapidly Screening Drought Tolerance in Potato

**DOI:** 10.1002/cpz1.70180

**Published:** 2025-07-28

**Authors:** Muhammad Awais Zahid, Nam Phuong Kieu

**Affiliations:** ^1^ Department of Plant Protection Biology Swedish University of Agricultural Sciences Lomma Sweden

**Keywords:** abiotic stress screening, CRISPR, drought tolerance, high‐throughput phenotyping, potato (*Solanum tuberosum*)

## Abstract

Potato (*Solanum tuberosum* L.) yield is highly sensitive to drought stress, yet robust phenotyping methods for drought tolerance remain scarce. To address this challenge, we present a rapid, high‐throughput hydroponic assay designed as an efficient pre‐screening tool for evaluating potato cultivars and CRISPR‐edited lines. This protocol serves as a foundational screen, enabling researchers to identify the most promising genotypes before committing resources to more extensive soil or field trials. The system uses repurposed pipette tip boxes as low‐cost, scalable hydroponic units, making the method highly accessible. Over a five‐week period, plantlets are subjected to controlled 24‐hr osmotic stress with polyethylene glycol (PEG‐6000) and then assessed for resilience based on biomass, photosynthetic measurements, and visual recovery. This resource‐efficient assay provides a controlled environment to minimize experimental noise and has been successfully applied to characterize CRISPR‐edited potato lines. By providing a reproducible platform for initial evaluation, this protocol accelerates the selection pipeline for developing robust potato varieties for a changing climate. © 2025 The Author(s). Current Protocols published by Wiley Periodicals LLC.

**Basic Protocol 1**: Preparation of stem explants and *in vitro* rooting

**Basic Protocol 2**: Assembly and maintenance of tip‐box hydroponic units

**Basic Protocol 3**: PEG‐induced drought treatment and recovery

**Basic Protocol 4**: Post‐stress phenotypic, biomass, and photosynthetic analysis

## INTRODUCTION

Drought stress is the foremost abiotic factor limiting the productivity and yield of potato (*Solanum tuberosum* L.), a globally critical food crop (George et al., 2017). As climate change intensifies, the need for drought‐resilient cultivars is becoming increasingly urgent to ensure global food security (Raza et al., 2023). However, progress in developing these varieties is often constrained by the time and resources required for traditional field‐based trials. To accelerate the research pipeline, rapid and controlled‐environment assays are invaluable for the initial characterization of new genotypes (Mansoor & Chung, 2024). These preliminary screens enable early identification of promising candidates, ensuring that only the most resilient lines advance to more extensive field testing.

While controlled‐environment assays using polyethylene glycol (PEG) can simulate drought (Steuter et al., 1981), existing methods on agar or in large‐scale hydroponics have limitations in either control or scalability. This creates a critical gap for a methodology that can efficiently and affordably screen large numbers of genotypes to identify promising candidates at an earlier stage.

This protocol describes an innovative, miniaturized hydroponic system designed specifically to fill this gap, serving as a foundational screen within a tiered research pipeline. Positioned as a crucial first step, this assay is not a replacement for soil‐based validation but a tool to make the entire selection process more efficient. By repurposing ubiquitous pipette tip boxes, the method provides a low‐cost and highly scalable platform to rapidly evaluate diverse cultivars and CRISPR‐edited lines in a small footprint. This approach will allow culling of sensitive genotypes and prioritizing the most resilient candidates for more demanding pot experiments and field trials. The method's utility has been successfully validated in published studies. For example, it was used to reveal that CRISPR/Cas9‐mediated knockout of the susceptibility gene *StDMR6‐1* in the ‘King Edward’ potato cultivar conferred enhanced tolerance to drought stress (Karlsson et al., 2024). Similarly, the assay demonstrated that deleting the novel gene *Parakletos* in the ‘Désirée’ cultivar resulted in enhanced drought tolerance (Zahid et al., 2024). These examples highlight the protocol's utility for the functional validation of candidate genes and essential early‐stage characterization of genome‐edited lines.

**Figure 1:**
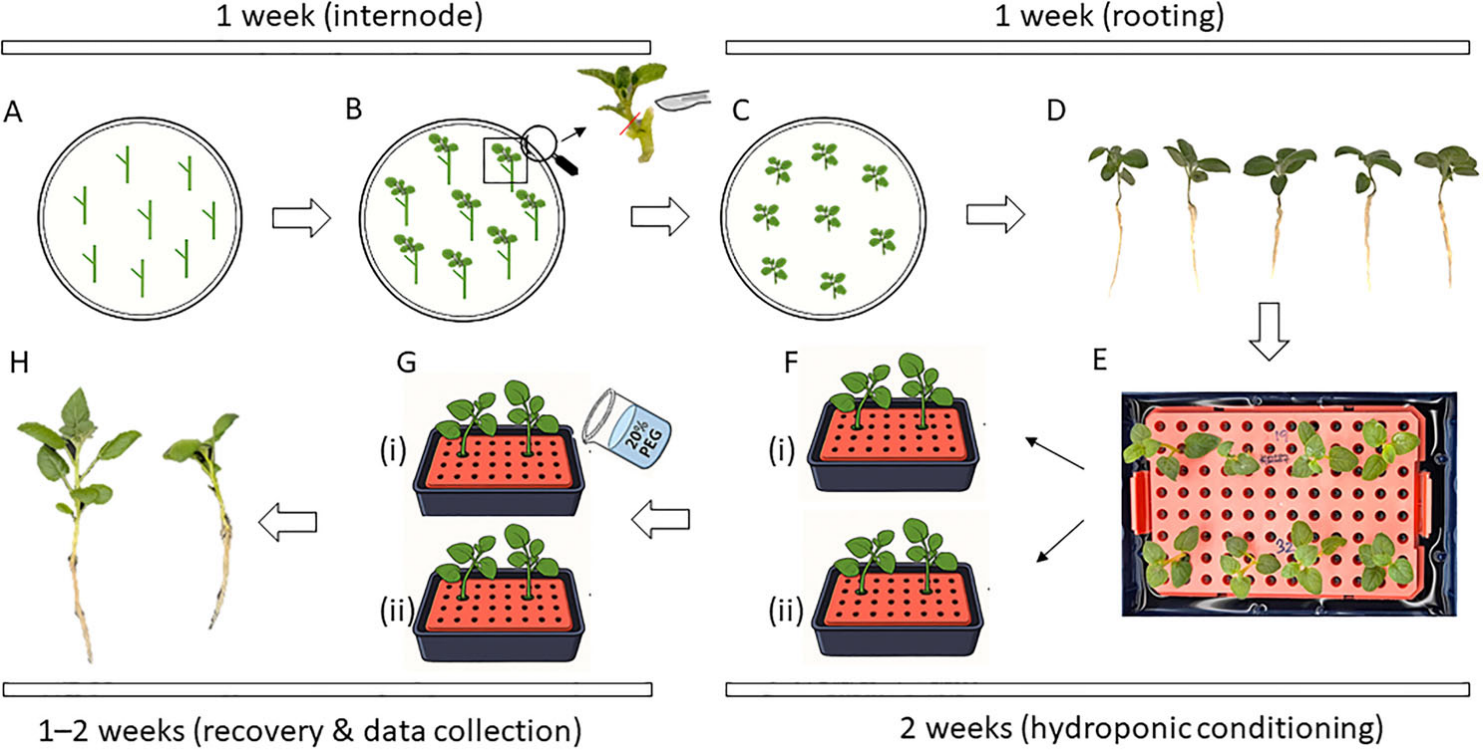
Schematic workflow of the rapid drought screening assay. (**A**) Potato internode cuttings are cultured on MS‐agar medium. (**B**) After 7–10 days, shoot tips emerge and are excised. (**C and D**) The shoot tips are rooted on fresh MS‐agar medium for 7 days to produce uniform plantlets. (**E**) Plantlets are transferred to the tip‐box hydroponic system for a pre‐conditioning period. (**F and G**) Plants are separated into control and stress groups; the stress group is exposed to 20% PEG‐6000 for 24 hr, followed by a recovery period. (**H**) At the end of the experiment, phenotypic and quantitative data, such as biomass and photosynthetic efficiency, are collected.

The following protocols provide a step‐by‐step guide for implementing this screening assay (Fig. 1). Basic Protocol 1 details the generation of uniform, axenic plantlets from stem cuttings *in vitro* to ensure consistent starting material. Basic Protocol 2 describes the assembly and maintenance of the low‐cost, tip‐box hydroponic units. Basic Protocol 3 outlines the procedure for applying the PEG‐induced osmotic stress and managing the subsequent recovery phase. Finally, Basic Protocol 4 provides instructions for data collection, including visual phenotyping, biomass measurement, and photosynthetic parameter analysis.

*CAUTION*: PEG‐6000 solution is highly viscous and can create slippery work surfaces; place absorbent, non‐slip mats around benches and immediately wipe spills.

*CAUTION*: Sterilize all media and perform all transfers in a laminar flow hood to maintain axenic culture conditions and prevent microbial contamination.

*CAUTION*: When working with genetically modified organisms (GMOs), such as the CRISPR‐edited lines that can be evaluated with this protocol, follow all appropriate institutional and national guidelines and regulations.

## STRATEGIC PLANNING

A well‐planned experiment is essential for obtaining robust and reproducible results. This protocol is designed to be completed in approximately 5 weeks, from the initial cutting to final data collection. For each genotype, include both a control group and a stress group, with a minimum of 3–4 boxes (12–18 plants each) per group.

Timeline:

Day 0: Prepare MS‐agar plates and place internode cuttings from donor plants on the medium to induce shoot formation (Fig. 1A).

Days 1–7: Incubate cuttings to allow for shoot emergence (Fig. 1B).

Day 8: Excise uniform shoot tips and transfer them to fresh MS‐agar plates for rooting (Fig. 1C).

Days 8–14: Incubate the shoot tips to allow for adequate root development, generating uniform plantlets (Fig. 1D).

Day 15: Select and transfer uniformly rooted plantlets to the tip‐box hydroponic system containing liquid MS medium (Fig. 1E).

Days 15–21: Grow plants in the hydroponic system for a pre‐conditioning period. Change the medium every 2–3 days.

Day 21‐28: During the pre‐conditioning period, randomly assign the uniform plantlets to the hydroponic boxes to either a “control” or “stress” group (Fig. 1F).

Day 29: Initiate the 24‐hr drought stress treatment by replacing the MS medium in the “stress” group boxes with 20% PEG solution (Fig. 1G).

Day 30: Remove the PEG solution from the stress group, wash roots thoroughly, and place all plants in fresh MS medium to begin the recovery phase.

Days 30–36: Monitor plant recovery, continuing to change the medium for all boxes every 2–3 days.

Day 38: Terminate the experiment. Take final photographs for visual assessment and harvest plants to measure biomass for quantitative analysis (Fig. 1H).

## Basic Protocol 1 PREPARATION OF STEM EXPLANTS AND *IN VITRO* ROOTING

This initial protocol is a critical preparatory stage focused on generating the high‐quality, uniform starting material that is foundational to a reproducible assay. The primary objective is to produce a cohort of axenic and uniform potato plantlets. Utilizing sterile *in vitro* material is paramount, as it eliminates confounding variables common to greenhouse or field‐grown plants, such as pre‐existing microbial loads, which can significantly impact experimental outcomes. The protocol employs a deliberate two‐step process: first, new shoots are induced from the axillary buds of tissue‐cultured internode cuttings, and second, these newly formed shoots are excised and rooted on an agar medium (Fig. 1A–D). It is important to note that rooting efficiency may differ between potato cultivars. A successful execution of this protocol will yield homogenous plantlets with well‐developed root systems (≥1 cm) and vigorous, green shoots, providing the ideal starting material for the subsequent transition to liquid culture.

### Materials

Stem internode segments (∼1 cm) from healthy, sterile donor plants
Murashige & Skoog (MS) basal salt mixture including vitamins, plant tissue culture grade (e.g., Duchefa Biochemie, cat. no. M0222)
Sucrose (e.g., Sigma‐Aldrich, cat. no. S0389 or Duchefa, cat. no. S0809)
Gelrite (e.g., Duchefa, cat. no. G1101)
Potassium hydroxide (KOH)
Sterile deionized water

Laminar flow hood, certified
Autoclave
Glass beakers and media bottles
Magnetic stirrer and stir bars
pH meter
Petri dishes (90 mm, sterile)
Forceps and scalpels (sterile)
3M micropore tape
Growth chamber or tissue culture room (22°C, 16 hr light/8 hr dark photoperiod, light intensity of ∼60 µmol m⁻² s⁻¹)

1 In a glass beaker, dissolve 4.4 g MS basal salts and 10 g sucrose per liter of deionized water using a magnetic stirrer.
2 Adjust the pH of the solution to 5.7 ± 0.1 using KOH.
3 Add 2.5 g Gelrite per liter.
4 Autoclave the medium at 121°C for 20 min.
5 Allow the medium to cool to approximately 50°C to 60°C in a water bath or on the bench. Pouring the medium while it is too hot can cause excessive condensation on the petri dish lids.
6 In a laminar flow hood, pour approximately 25 ml of the medium into each sterile petri dish.
7 Leave the plates partially open in the hood for 15–20 min to solidify and allow condensation to evaporate, then close and store them until use.
8 Excise several stem internode segments (∼1–2 cm long) from healthy donor plants.
9 Using sterile forceps, place 7–8 sterilized internode segments horizontally onto the surface of the MS‐agar in each petri dish under a laminar flow hood (Fig. 1A).
10 Seal the plates with 3M micropore tape and incubate in the growth chamber for 7–10 days to allow for the emergence of new axillary shoots (Fig. 1B).
11 Select vigorous, healthy shoots of approximately 1– 2 cm in length. Under a laminar flow hood, use a sterile scalpel to excise these shoots from the original internode cutting.
12 Transfer the excised shoot tips onto fresh MS‐agar plates, planting them vertically with the cut end inserted about 0.5 cm into the medium (Fig. 1C). Incubate the plates for another 7 days to allow for root development.
13 Select plantlets that have developed a healthy root system (≥1 cm) and uniform shoot growth for the next stage (Fig. 1D). Discard any non‐uniform or poorly rooted plantlets to ensure a homogenous experimental group. Proceed with the selected plantlets to the next protocol.

## Basic Protocol 2 ASSEMBLY AND MAINTENANCE OF TIP‐BOX HYDROPONIC UNITS

**Figure 2:**
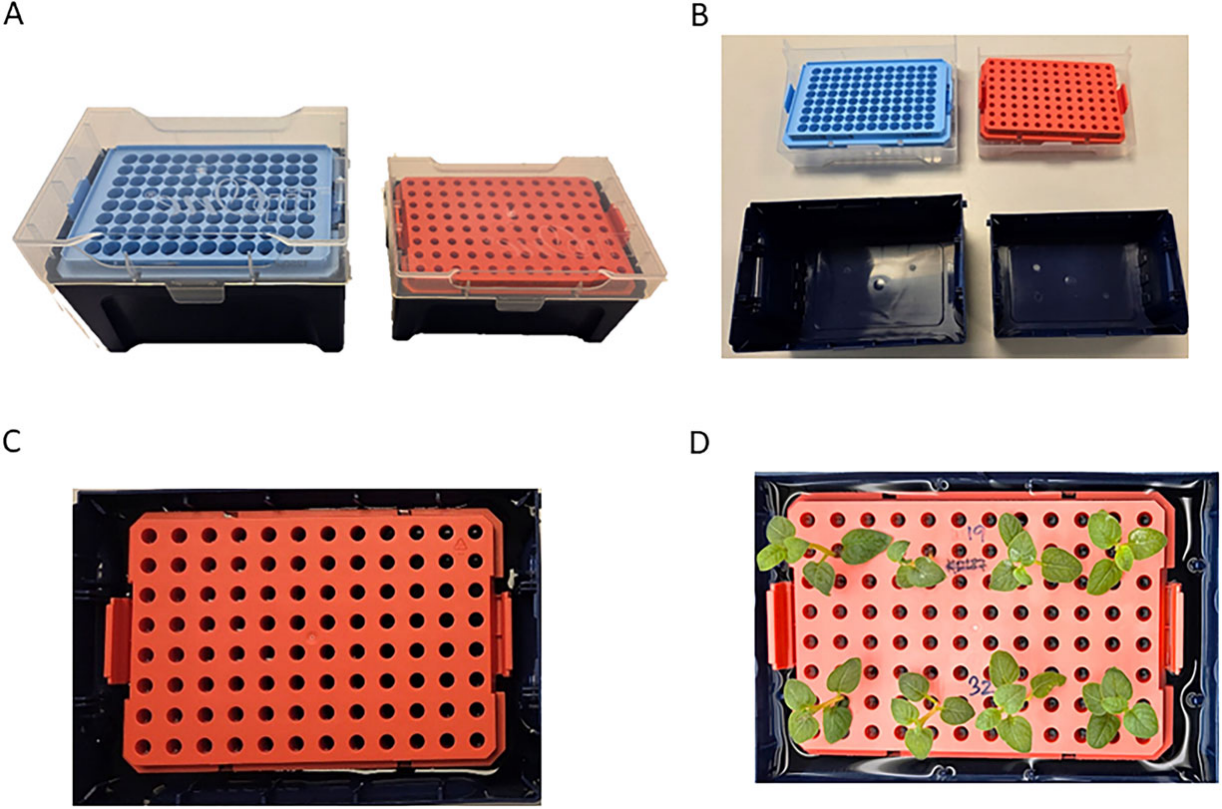
Assembly and setup of the tip‐box hydroponic unit. (**A**) The components of the hydroponic unit are a standard 1‐ml pipette tip box (bottom) and a 10/20 µl tip box (top, with rack). (**B and C**) The 1‐ml box serves as a reservoir for the liquid MS medium, while the inverted 10/20‐µl rack serves as a support for the plantlets. (**D**) A fully assembled unit showing potato plantlets with their shoots supported by the rack and roots submerged in the nutrient solution.

This protocol details the assembly of the “tip‐box” hydroponic system and the crucial pre‐conditioning of the plantlets (Fig. 2). The core of this step is the conversion of inexpensive, readily available pipette tip boxes into functional, autoclavable culture vessels. The purpose of this stage is twofold: first, to establish the physical setup, and second, to acclimate the young plantlets to a new environment. Transferring plants from solid agar to a liquid medium requires a pre‐conditioning period for the root system to adapt its morphology and physiology for nutrient uptake directly from a solution. Properly executing this 14‐day phase ensures that all plants are physiologically stable and actively growing before stress is imposed, which is essential for obtaining consistent and reliable results in the subsequent drought assay.

### Materials

Rooted potato plantlets (from Basic Protocol 1)
Liquid MS medium (see recipe)

Tap water

Autoclavable 1‐ml pipette tip boxes (bottom reservoir) (Fig. 2A)
Autoclavable 10/20‐µl pipette tip boxes (with rack for plant support) (Fig. 2)
Autoclave
Sterile beakers
Sterile forceps
Growth chamber or tissue culture room (22°C, 16 hr light/8 hr dark photoperiod, light intensity of ∼120 µmol m⁻² s⁻¹)

1 Take a 1‐ml pipette tip box (the reservoir) and a 10/20‐µl tip box. Remove the lid from the 10/20 µl box and invert the rack containing the tip holders (Fig. 2A‐B).
2 Place the inverted rack on top of the 1 ml box reservoir to create the hydroponic unit (Fig. 2C).
3 Loosely cover the assembled units with aluminum foil and autoclave them at 121°C for 20 min. Autoclaving the pre‐assembled units minimizes handling and the risk of algal growth.
4 Pour approximately 500–700 ml liquid MS medium (pH 6.0) into each autoclaved reservoir.
5 Gently remove the rooted plantlets from the agar plates. To remove residual agar, swirl each plantlet's root system in a beaker of water. Avoid pulling or rubbing the delicate roots.
6 Using sterile forceps, carefully thread the root system of one plantlet through each aperture in the tip rack. Ensure the roots are fully submerged in the liquid medium while the crown of the plant rests on top of the rack (Fig. 2D).
7 Place the fully assembled hydroponic units in the growth chamber and grow the plants for a 14‐day pre‐conditioning period (Fig. 1E).
8 Replace the liquid MS medium every 2–3 days (e.g., Monday, Wednesday, Friday). To do this, lift the plant support rack, discard the old medium, and refill the reservoir with fresh medium. This regular change is critical to replenish nutrients. At the end of this period, select the most uniform and healthy boxes for the stress experiment.

## Basic Protocol 3 PEG‐INDUCED DROUGHT TREATMENT AND RECOVERY

**Figure 3:**
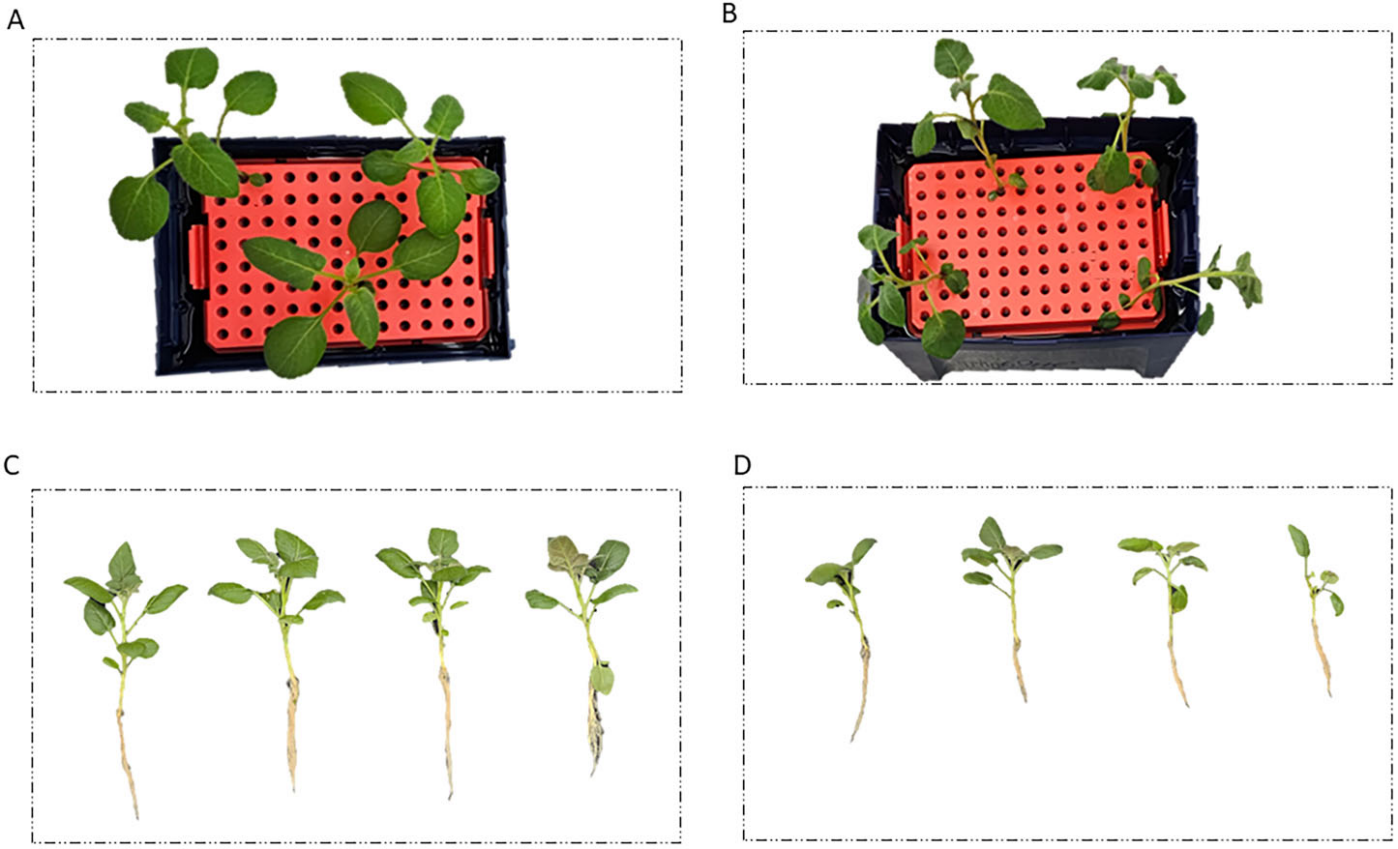
Representative visual phenotypes during stress and recovery. (**A and B**) Comparison of potato plantlets at the end of the 24‐hr treatment period. The control plant (**A**) remains healthy, while the stressed plant (**B**) exhibits severe wilting. (**C and D**) Differential phenotypes after the recovery period. The control group (**C**) shows vigorous growth, while the plantlets subjected to stress (**D**) show significant growth inhibition and reduced biomass.

This protocol constitutes the core experimental treatment of the screening assay, where controlled and drought stress are applied. The method uses a 24‐hr exposure to 20% polyethylene glycol (PEG‐6000) to induce severe osmotic stress. This acute stress pulse is designed to be strong enough to reveal key differences in tolerance, observable by differences in wilting (Fig. 3B), yet transient enough to allow for the assessment of recovery potential. The ability to recover after stress is often a more critical indicator of field resilience than the response during stress itself. Therefore, the subsequent step, thoroughly rinsing the PEG from the root systems before returning plants to fresh nutrient medium, is absolutely critical for an accurate assessment.

### Materials

Pre‐conditioned potato plants in hydroponic units (from Basic Protocol 2)
20% (w/v) PEG‐6000 solution in liquid MS (see recipe)
Liquid MS medium (see recipe)

Tap water in large beakers (for rinsing)

Gloves
Waste container for PEG disposal

1 On day 28, at the end of the pre‐conditioning period, arrange all hydroponic boxes by genotype.
2 Within each genotype, assign boxes to ‘control’ or ‘stress’ groups (Fig. 1F).
3 Working with one box at a time, carefully lift the plant support rack. For the “stress” group, decant the MS medium and replace it with 500–700 ml of the 20% PEG solution (Fig. 1G).
  Verify that the roots are properly submerged in the solution.
4 For the “control” group, replace the old MS medium with an equal volume of fresh liquid MS medium.
5 Return all boxes to the growth chamber and incubate for 24 hr.
  After several hours, a clear wilting phenotype should be visible in the stressed plants (Fig. 3B), while control plants remain turgid (Fig. 3A). This serves as a visual confirmation that the stress is effective.
6 After the 24‐hr stress period, remove the plant racks from the PEG solution.
7 To rinse the roots, gently dip and swirl the entire rack of plants in water for 30 s.
8 Repeat this rinsing process at least three more times, each time in a fresh beaker of sterile water.
  This step is critical. Insufficient rinsing will leave a PEG residue that inhibits water uptake and will confound the recovery results.
9 Place the rinsed plant racks back into their corresponding reservoirs, now filled with fresh liquid MS medium.
10 Grow all plants (control and stress groups) for an additional 9 days (until day 38), continuing to renew the medium for all boxes every 2–3 days to support recovery and growth.

## Basic Protocol 4 POST‐STRESS PHENOTYPIC, BIOMASS, AND PHOTOSYNTHETIC ANALYSIS

**Figure 4:**
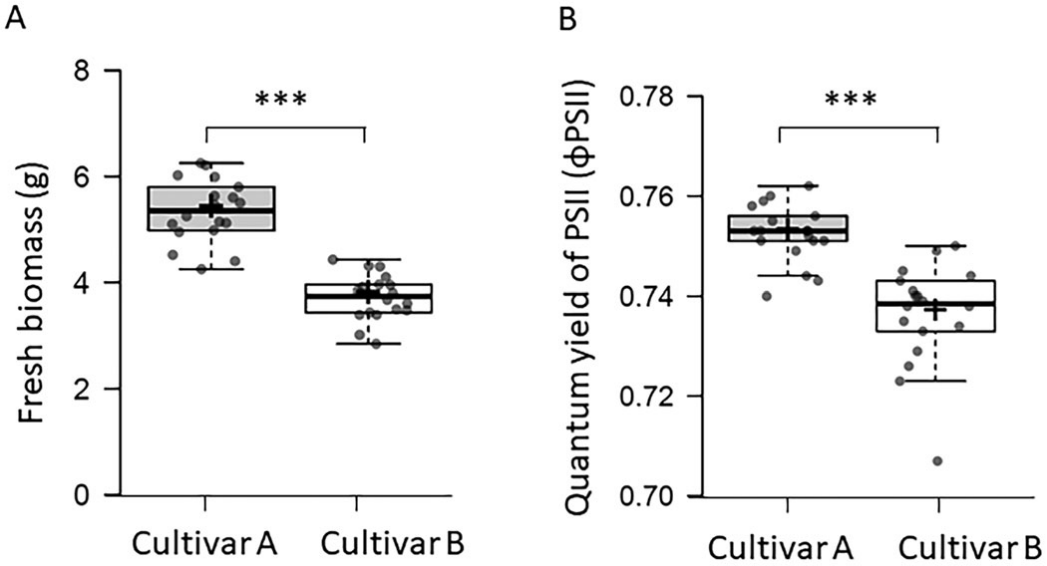
**Quantitative analysis of potato plantlets after recovery**. Box plots comparing the physiological and growth responses of two potato cultivars (Cultivar A and Cultivar B) after the recovery period. (**A**) Fresh biomass (g). (**B**) Quantum yield of PSII (φPSII). Data represent the mean of n = 18 plants. The centerline in the box plots indicates the median, the '+' sign indicates the mean, the box borders delimit the lower and upper quartiles, and the whiskers show the full data range. Asterisks indicate a statistically significant difference between cultivars (Student's *t*‐test, ***p < 0.0001).

This final protocol outlines the data collection and quantification steps needed to evaluate plant performance after the recovery period. A robust assessment of drought tolerance relies on integrating multiple data types, and this protocol combines qualitative visual documentation with quantitative biomass and photosynthetic efficiency measurements. Visual assessment (Fig. 3C and 3D) is important for capturing key phenotypic traits that are not reflected in biomass alone. This is complemented by measuring the fresh weight, which reflects the plant's ability to maintain water balance, and the quantum yield of Photosystem II (PSII), a direct indicator of photosynthetic health. Drought stress reduces the quantum yield of PSII, and measuring this parameter provides a rapid, non‐invasive assessment of the plant's physiological status (Chen et al., 2016; Colom & Vazzana, 2003; Sperdouli & Moustakas, 2012). By comparing these metrics between cultivars or gene‐edited lines, a clear, statistically defensible ranking of genotypes can be achieved (Fig. 4), enabling the confident selection of the most resilient lines for further study.

### Materials

Handheld fluorometer (e.g., MultispeQ)
Digital camera with a tripod and a consistent lighting setup
Neutral background (black or white) for photography
Analytical balance (±1 mg)
Paper towels
Drying oven with air circulation (60°C)
Labeled weighing boats or aluminum foil envelopes

1 On day 37 (approximately 1 week into the recovery period), measure the photosynthetic efficiency of the plants.
  Do not take these measurements immediately after the 24‐hr stress period, as severe wilting can interfere with obtaining reliable data. A 1‐week recovery period allows for physiological stabilization.
2 Use a handheld fluorometer (e.g., MultispeQ (Kuhlgert et al., 2016)) to record the Quantum Yield of PSII (φPSII) from the youngest fully expanded leaf of each plant in both the control and stress groups.
  For consistent results, take measurements from the same leaf position on all plants and at the same time of day to minimize diurnal variation. Since this measurement is non‐destructive, it can be taken multiple times during the recovery phase (e.g., at 3, 5, and 7 days after stress removal) to create a recovery time.
3 On day 38, at the end of the recovery period, photograph each hydroponic box against a neutral background. Maintain a consistent camera distance, angle, and lighting for all photos to allow for a fair visual comparison of the final recovery status.
4 Working with one box at a time, carefully remove the plant support rack.
5 Remove each plant individually and photograph the whole plant (shoots and roots) against a neutral background to document individual morphology (compare Fig. 3C and 3D).
6 Gently blot the entire plant with paper towels to remove all surface moisture.
  Apply consistent pressure and blotting duration for each plant to ensure comparable measurements.
7 Immediately place the blotted plant on the analytical balance and record its fresh weight (FW).
8 Collate the fresh weight (FW) and quantum yield of PSII (φPSII) data for all plants from both control and stress groups.
9 Visualize the data distribution for each group using box plots (Spitzer et al., 2014). In the plots, the centerline indicates the median, the ‘+’ sign indicates the mean, the box borders delimit the lower and upper quartiles, and the whiskers show the highest and lowest data points (see Fig. 4 for an example).
10 Analyze the data set using appropriate statistical software to test for significant differences between treatments and genotypes. A two‐way ANOVA is suitable for testing the effects of genotypes and treatment, followed by a post‐hoc test for pairwise comparisons.
11 The resulting data provides a comprehensive evaluation of genotype performance, enabling the selection of promising candidates for further validation in soil‐based experiments.

## Reagents and Solutions

### Liquid MS medium

4.3 g/5 L Murashige & Skoog basal salts (e.g., Duchefa, cat. no. M0221)
Dissolve in tap water. Adjust pH to 6.0 ± 0.1 with KOH.
Store up to 3 months at 2–8°C.

### MS‐agar medium

4.4 g/L Murashige & Skoog basal salts including vitamins (e.g., Duchefa, cat. no. M0222)
10 g/L Sucrose
2.5 g/L Gelrite
Dissolve in deionized water. Adjust pH to 5.7 ± 0.1 with KOH. Autoclave at 121°C for 20 min. Pour into sterile petri dishes in a laminar flow hood.
Store at 2–8°C and use within 2–4 weeks.

### PEG‐6000 Solution, 20% (w/v)

200 g PEG‐6000 (e.g., Sigma‐Aldrich, cat. no. 81260)
Dissolve in 800 ml tap water, warming to 60°C with stirring if necessary to fully dissolve.
Bring the final volume to 1 L with Liquid MS medium. Adjust pH to 6.0.
Autoclave at 121°C for 20 min and cool to room temperature before use.
Must be refrigerated if stored long‐term (−20°C or 4°C).

## Commentary

### Critical Parameters

#### Quality and uniformity of plant material

This is the most critical factor for success. The protocol begins with in vitro propagation to ensure all plantlets are axenic, uniform, and at a similar developmental stage. When selecting shoots for rooting and later for transfer to hydroponics, rigorously discard any outliers in size, vigor, or root development. Starting with a non‐uniform population will lead to high variability in the final data, making it difficult to detect true genetic differences.

#### Health of donor plants

The physiological state of the initial donor plants used for explants can influence the outcome. Use material from healthy, well‐nourished plants that are in an active state of growth. Cuttings from stressed or senescing plants may exhibit poor rooting and reduced vigor in vitro.

#### PEG solution and rinsing procedure

The concentration and application of the PEG solution must be precise. The solution should be completely dissolved and cooled to room temperature before use to avoid temperature shock. The post‐stress rinsing procedure is one of the most important steps; the viscous PEG solution clings to roots and, if not completely removed, will continue to exert osmotic stress, preventing true recovery and obscuring the results. Ensure a minimum of four vigorous rinses in fresh, sterile water.

#### Growth chamber environment

Maintain a consistent environment throughout the experiment. Fluctuations in light intensity, temperature, or photoperiod can introduce variability. The recommended light intensity is suitable for healthy growth. For tissue culture plants, light intensity should be ∼60 µmol m^−^² s⁻¹ and for a hydroponic system, it needs to be ∼120 µmol m⁻² s⁻¹. Higher intensities can exacerbate photo‐oxidative damage during the stress and recovery phase, confounding the results.

#### Contamination control

A multi‐layered strategy is essential to prevent microbial contamination, which is managed through media composition, initial sterility, environmental control, and regular maintenance. First, the liquid MS medium used for the hydroponic stage is a simple basal salt solution without added vitamins or a carbon source (e.g., sucrose). This minimal‐nutrient formulation is a primary deterrent, as it cannot support the significant growth of heterotrophic microbes such as fungi and most bacteria. Second, the protocol begins with axenic plantlets and uses autoclaved hydroponic units to ensure clean starting conditions. Third, the entire assay must be conducted within a tissue culture laboratory or an equivalent cleanroom. This is a critical prerequisite, as the low ambient level of airborne spores in such an environment minimizes inoculum pressure. Finally, the liquid medium is renewed every 2–3 days. This regular flushing physically removes any potential contaminants before they can proliferate. The most common contaminant in this system is algae; if this becomes an issue, we recommend performing plant transfers in a laminar flow hood for enhanced sterility.

#### Consistency in measurements

All measurements on the final day should be performed with high consistency. When measuring photosynthetic efficiency (φPSII), always select the same leaf position (e.g., the youngest fully expanded leaf) and take readings at the same time of day to avoid effects from circadian rhythms. When blotting plants for fresh weight, the duration and pressure should be standardized for all samples.

### Troubleshooting Table

Table 1 outlines common problems that may be encountered, their likely causes, and potential solutions.

**Table 1:** Troubleshooting Guide

Problem	Possible cause	Solution
Poor rooting of explants	Donor plant health was poor; explants were woody or senescent.	Use young, actively growing stems from healthy donor plants. Add a low concentration of auxin (e.g., 0.1 mg L⁻¹ IBA) to the rooting medium if needed. Donor plants can be refreshed as well (Wang et al., 2020).
Algal or microbial contamination	Improper sterile technique; incomplete autoclaving.	Autoclave hydroponic units fully assembled; check autoclave performance.
High variability in plant growth	Non‐uniform starting plantlets; uneven light/temp.	Rigorously select uniform plants at each stage; use a randomized block design and rotate boxes within the growth chamber weekly.
Control plants appear stressed/yellow	Nutrient depletion in the hydroponic solution.	Ensure the liquid MS medium is changed regularly (at least 3 times per week), especially as plants increase in size.
All plants die after PEG stress	PEG concentration too high for the tested genotypes; incomplete rinsing.	Test a lower PEG concentration (e.g., 15%) or treat them in PEG overnight instead of 24 hr; ensure roots are rinsed thoroughly (at least 4 times) with fresh water.
Inconsistent fluorescence readings	Diurnal variation, inconsistent leaf selection, or age.	Take all φPSII measurements at the same time of day; consistently measure the same leaf position (e.g., youngest fully expanded leaf) on all plants.
Roots turn brown in hydroponics	Poor aeration or potential microbial contamination	Ensure the nutrient solution level is not too high, allowing some roots access to air. If contamination is suspected, discard the affected box.

### Understanding Results

A successful experiment will yield clear, quantifiable differences between genotypes, allowing for a robust assessment of their recovery potential. The interpretation should integrate all collected data points.

#### Visual phenotypes

The photographic record is a powerful tool (Fig. 3). A tolerant genotype will typically show a rapid recovery of turgor after being returned to fresh medium, minimal leaf necrosis, and the initiation of new growth. A sensitive genotype will often fail to regain turgor, exhibit progressive leaf yellowing and death, and show little to no new growth.

#### Quantitative data interpretation

The primary quantitative metrics are fresh weight (FW) and the quantum yield of PSII (φPSII) (Fig. 4). The most informative way to compare genotypes is by calculating the percent retention for each metric relative to the unstressed controls. A tolerant line will exhibit a high percent retention of both FW and φPSII, indicating it was able to maintain its water balance and quickly repair its photosynthetic machinery. A sensitive line will show low retention values.

#### Interpreting combined data

Consider the relationship between the metrics. For example, a plant that retains high FW but has very low φPSII may have maintained its water status but suffered severe, lasting damage to its photosystems. Conversely, a plant with lower FW retention but a rapidly recovering φPSII may be exhibiting a strategy of shedding older leaves to prioritize the health of new growth. These combined insights provide a more nuanced understanding of a plant's specific resilience strategy.

#### Translating to field performance

It is crucial to understand that this protocol assays the response to a single, acute osmotic stress event in a controlled environment. While it is a powerful tool for pre‐screening and functional validation, a high rank in this assay does not guarantee superior performance under all field conditions, which involve complex, fluctuating stresses (e.g., chronic drought, heat, pests). The results from this foundational screen should be used to make informed decisions about which promising genotypes warrant advancement to more complex and resource‐intensive pot and field trials for final validation.

### Time Considerations

The entire protocol, from preparing explants to final data collection, takes approximately 38 days (about 5 weeks). The hands‐on time is relatively low, estimated at around 8–10 hr spread over the entire period. The most time‐consuming steps are the initial explant preparation and the final harvest and data collection day. Routine work, such as changing the medium, requires about 30 min, three times per week.

### Author Contributions

**Muhammad Awais Zahid**: Conceptualization; funding acquisition; investigation; methodology; project administration; resources; visualization; writing—original draft; writing—review and editing. **Nam Kieu Phuong**: Conceptualization; funding acquisition; investigation; methodology; writing—review and editing.

### Conflict of Interest

The authors declare no conflict of interest.

## Data Availability

The data, tools, and material (or their source) that support the protocol are available from the corresponding author upon reasonable request.
